# A Novel Approach to Assess Sleep-Related Rhythmic Movement Disorder in Children Using Automatic 3D Analysis

**DOI:** 10.3389/fpsyt.2019.00709

**Published:** 2019-10-16

**Authors:** Markus Gall, Bernhard Kohn, Christoph Wiesmeyr, Rachel M. van Sluijs, Elisabeth Wilhelm, Quincy Rondei, Lukas Jäger, Peter Achermann, Hans-Peter Landolt, Oskar G. Jenni, Robert Riener, Heinrich Garn, Catherine M. Hill

**Affiliations:** ^1^Sensing and Vision Solutions, AIT Austrian Institute of Technology GmbH, Vienna, Austria; ^2^Sensory-Motor Systems Lab, ETH Zurich, Zurich, Switzerland; ^3^Sleep & Health Zurich, University Center of Competence, University of Zurich, Zurich, Switzerland; ^4^Institute for Pharmacology and Toxicology, University of Zurich, Zurich, Switzerland; ^5^The KEY Institute for Brain Mind Research, Department of Psychiatry, Psychotherapy and Psychosomatics, University Hospital of Psychiatry, Zurich, Switzerland; ^6^Child Development Center, University Children’s Hospital Zurich, Zurich, Switzerland; ^7^Faculty of Medicine, University of Zurich, Zurich, Switzerland; ^8^Division of Clinical and Experimental Sciences, Faculty of Medicine, University of Southampton, Southampton, United Kingdom

**Keywords:** 3D video, automated, rhythmic movement disorder, contactless, Jacinto capita nocturna, diagnostic tool, sleep

## Abstract

**Background:** Unlike other episodic sleep disorders in childhood, there are no agreed severity indices for rhythmic movement disorder. While movements can be characterized in detail by polysomnography, in our experience most children inhibit rhythmic movement during polysomnography. Actigraphy and home video allow assessment in the child’s own environment, but both have limitations. Standard actigraphy analysis algorithms fail to differentiate rhythmic movements from other movements. Manual annotation of 2D video is time consuming. We aimed to develop a sensitive, reliable method to detect and quantify rhythmic movements using marker free and automatic 3D video analysis.

**Method:** Patients with rhythmic movement disorder (n = 6, 4 male) between age 5 and 14 years (M: 9.0 years, SD: 4.2 years) spent three nights in the sleep laboratory as part of a feasibility study (https://clinicaltrials.gov/ct2/show/NCT03528096). 2D and 3D video data recorded during the adaptation and baseline nights were analyzed. One ceiling-mounted camera captured 3D depth images, while another recorded 2D video. We developed algorithms to analyze the characteristics of rhythmic movements and built a classifier to distinguish between rhythmic and non-rhythmic movements based on 3D video data alone. Data from 3D automated analysis were compared to manual 2D video annotations to assess algorithm performance. Novel indices were developed, specifically the rhythmic movement index, frequency index, and duration index, to better characterize severity of rhythmic movement disorder in children.

**Result:** Automatic 3D video analysis demonstrated high levels of agreement with the manual approach indicated by a Cohen’s kappa >0.9 and F1-score >0.9. We also demonstrated how rhythmic movement assessment can be improved using newly introduced indices illustrated with plots for ease of visualization.

**Conclusion:** 3D video technology is widely available and can be readily integrated into sleep laboratory settings. Our automatic 3D video analysis algorithm yields reliable quantitative information about rhythmic movements, reducing the burden of manual scoring. Furthermore, we propose novel rhythmic movement disorder severity indices that offer a means to standardize measurement of this disorder in both clinical and research practice. The significance of the results is limited due to the nature of a feasibility study and its small number of samples. A larger follow up study is needed to confirm presented results.

## Introduction

Rhythmic movement disorder (RMD) is a poorly understood sleep-related movement disorder defined by the International Classification of Sleep Disorders (ICSD-III) (1) as repetitive, stereotyped rhythmic movements (RMs) of large muscle groups in the frequency range 0.5–2 Hz (1), (2). The predominant forms are head-banging, body-rocking, and head-rolling (3). Typically, RMD starts in infancy with a maximum prevalence rate of 2.87% as confirmed by our recent community-based study of 1,447 infants and toddlers (4).

A diagnosis of RMD is only made when there are clinical consequence of nocturnal movements, specifically significant sleep disturbance, impaired daytime functioning, or physical injury (1). While RMD may cause local trauma and hair loss (5), reports of more serious injuries are rare (6). RMD can have social consequences; it may cause embarrassment (6, 7) and noise from head-banging or movements of the bed may disturb other household members (8). Importantly however, RMD can significantly affect sleep quality. Limited studies report poor concentration, difficult behavior, as well as impaired memory and decision making capabilities in children with this condition (9, 10).

The American Academy of Sleep Medicine (AASM) provides rules for scoring RMs using polysomnography. An episode of RMs consists of at least four movements, occurring with a frequency range from 0.5 Hz to 2.0 Hz and electromyography (EMG) should be at least twice the baseline amplitude (11). Guidance on assessment of severity is not provided. This contrasts with other childhood sleep disorders, for example, sleep disordered breathing, where decades of research in large populations of children have refined diagnostic criteria, allowing associations to be made between a measure of severity, the apnea/hypopnea index (AHI), and meaningful outcomes (12). This is a critical omission for RMD as objective measures are essential to improve diagnosis, monitor treatment outcomes, and standardize research. Thus, an important next step in progressing our understanding of this disorder is an agreed measure of severity and a standardized and objective method to quantify movements.

Furthermore, our clinical experience shows that many children fail to exhibit their typical RMs when constrained by sensors in a sleep laboratory environment (3). Stepanova et al. (13) also noted that polysomnography underestimates RMs compared to parental report. Thus, in children with suspected RMD, polysomnography is primarily used to exclude other disorders that cause movements during sleep such as epilepsy and periodic leg movements and, where rhythmic movements are seen, to confirm the clinical diagnosis.

Another technology, actigraphy, successfully quantifies movement amplitude and sleep quality but fails to distinguish RMs from other high amplitude movements (14). Manual observation and annotation of standard two-dimensional (2D) videosomnography (overnight video alone) is another method to assess RMD with the advantage that children can be assessed over multiple nights in their home bedroom environment unconstrained by sensors. While simple digital film images of movements can confirm a clinical diagnosis, quantification of the timing, duration, and episodic nature of movements require time-consuming and intensive analysis. Despite these limitations, videosomnography is a promising area for future research (15). One approach to address these limitations is 3D video (3). Recent research has demonstrated the utility of contactless 3D video for the detection of abnormal chest wall movements in central sleep apnea syndrome (16) and limb movements in periodic limb movement (PLM) disorder (17). Using 3D has the advantage to also include the depth axis, not accessible using 2D video analyses alone.

In summary, standards for objective quantification and characterization of RMs are not well established and standard technologies are imperfect. We aimed to address these limitations by evaluating a novel automated 3D assessment algorithm to quantify RMs. Furthermore, we aimed to develop novel indexes to quantify RMD severity as a basis for future international standards of assessment in this poorly understood disorder.

## Material and Methods

### Setting

Data were collected as part of a feasibility study (https://clinicaltrials.gov/ct2/show/NCT03528096) of sensory stimulation therapy in children with RMD (18, 19). Assessments were made in the University of Zurich, sleep laboratory, Switzerland. The study was approved by the ethics committees in both the UK (National Research Ethics Committee and Health Research Authority - IRAS 234505) and the Cantonal Ethics Committee of Zurich (KEK 2017-01880) alongside approvals from Swissmedic (2017-MD-0035). Parents of participating children signed informed consent, and children signed informed assent.

Children were aged 5 to 18 years with a clinical diagnosis of RMD confirmed by a somnologist (CMH). Children were excluded if they were sensitive to motion sickness based on self-report. Parent report of RMD semiology, history of injury, influence on sleep, and daytime functioning were recorded.

#### Sleep Laboratory Setup

**Figure 1:**
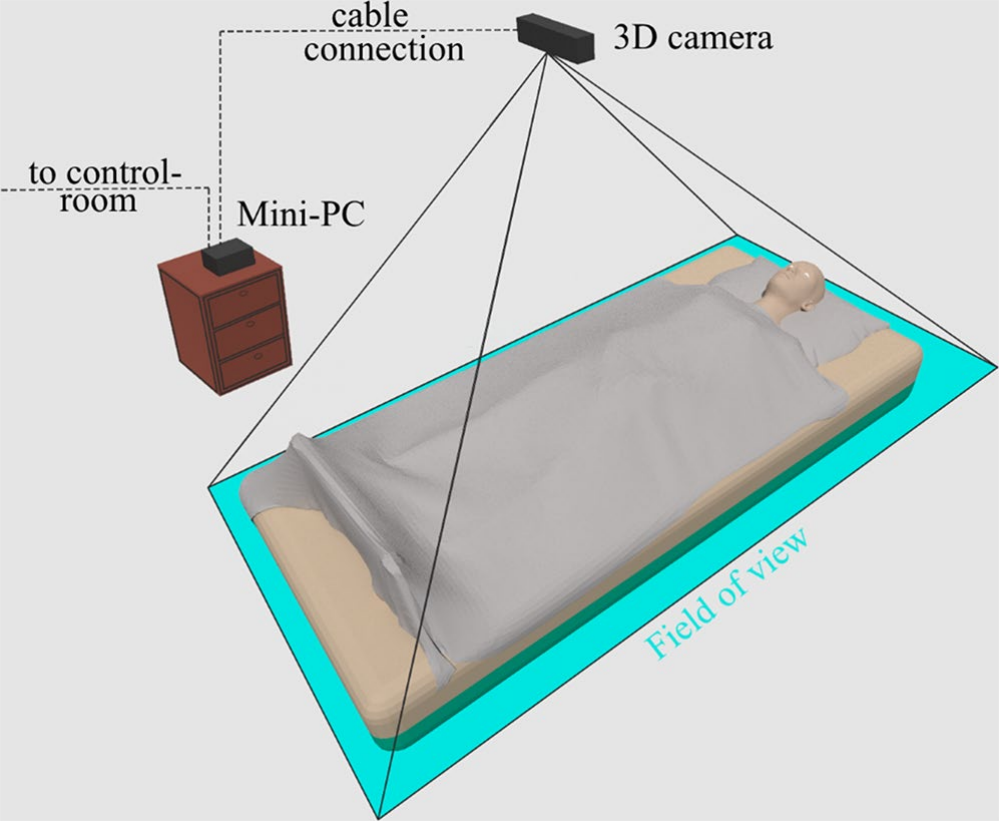
Sleep laboratory setup. The participant was placed in a bed with a 3D camera (Kinect v2) mounted on the ceiling. The camera was directed towards the participant with the field of view covering the bed area. The recorded video was transmitted to a fan-less mini-PC located in the same room. This PC further transmitted the data to a notebook located in the control room.

Rooms were equipped with hardware, as illustrated in **Figure 1**.

The participants slept one night in a standard bed (adaptation night) and another one in a bed that can rock gently (Somnomat) (18, 19) but was stationary for the measurement (baseline night). In both nights, Sheets were of average thickness. The feasibility study included a third night (intervention night) where the bed was rocking. However, this night was excluded from our 3D analysis since bed induced movements could not be distinguished from actual RMs reliably yet.

We used a time-of-flight sensor (Microsoft Kinect One V2) (20) as 3D camera mounted at the ceiling approximately 1.3 m above the bed to record 2D gray scale and 3D video. The time-of-flight principle measures the distance between the sensor and an object, based on the time it takes the infrared light from emission until its return to the sensor, after being reflected by an object. As a result, each pixel of the obtained 3D images holds the value of the distance to the nearest object detected. Further detail on the camera is available in the appendix section 9.1. The area of interest was set to the edges of the bed and thus created a depth map of the bed and participant. The sensor was mounted with a magnetic clip and additionally secured using a safety strap. A frame rate of 30 frames *per* second was used with radiation intensity compliant with current safety standards (21) for optical radiation.

The obtained 3D and 2D videos were transmitted to the control room using a fan-less mini-PC. A notebook equipped with sufficient storage capacity saved the data for further processing. Synchronization was automatically achieved between manual 2D and automatic 3D annotations using manually set, clear markers such as lights on/off.

### Definition of RMs and the Ground Truth

AASM recommendations for scoring an episode of RM, based on muscle activity recordings using electromyography sensors (22), were adapted to fit the method of visual scoring of 2D video:

- RM frequency was defined to be in the range of 0.5–2.0 Hz.
- The minimum number of movements required to define an episode of RMs was 4.

To define discrete episodes, the authors determined an additional criterion: if no movement occurred during a period of time equivalent to two RMs, the episode was scored as complete and any further movements immediately following this interval were defined as a new episode.

Scoring was performed manually by RS and EW. Episodes of RMs, and periods without movements, or with general non-RM, were annotated using start and duration entries. Movements were annotated while simultaneously monitoring experimental nights and offline revised to their best knowledge. This manual scoring approach formed the ground truth for 3D algorithm performance measure.

### 3D Algorithm

**Figure 2:**
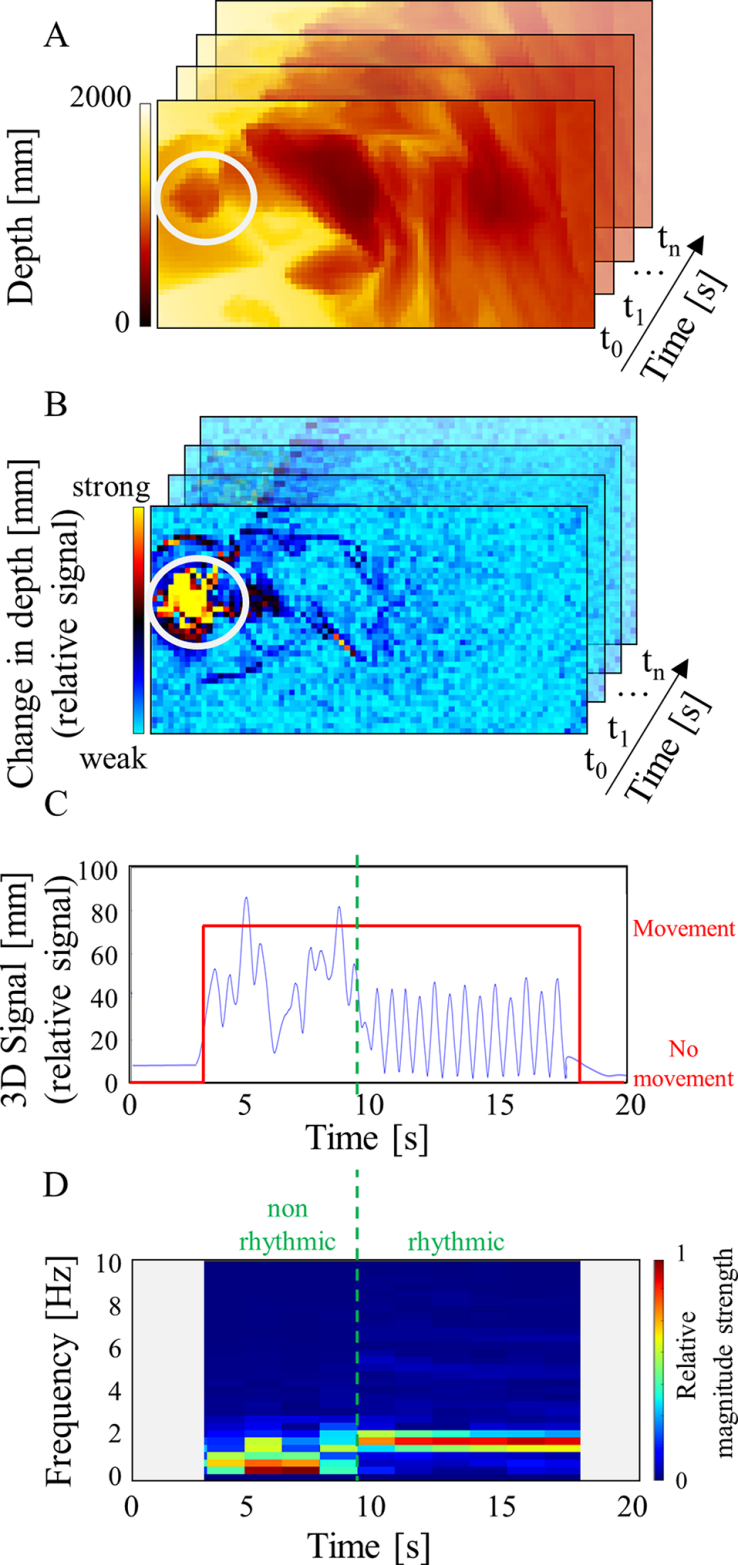
3D algorithm processing pipeline. The chosen example shows a child lying in a bed where the head is indicated in **(A** and **B)** by the white circle. **(A)** The pre-processed depth image stores the distance between camera and bed for each pixel in color coded millimeters. The example shows a person lying in bed covered with a blanket. A white circle highlights the participant’s head. **(B)** The motion map indicates movement. Here, a movement augmentation in the head region is present (yellow area). **(C)** The 3D signal (blue) shows a deflection for this time interval and a movement is annotated (red) where the 3D signal is augmented. Represented is a movement sequence where the first part reflects the participant changing from a lying position to a RM position on all fours followed by RMs. **(D)** The FFT spectrogram shows the frequencies over time. Relative strength of the magnitude is indicated by the colorbar. The RM part shows a narrower frequency band around 2Hz (right of green line). In contrast, the transitional movement shows a blurred spectrum (left of green line).

The 3D algorithm was an automated processing pipeline with the aim to detect movements and classify these into RMs and non-RMs based on raw 3D image input (**Figure 2**).

Firstly, obtained 3D images were pre-processed by subsampling and noise reduction using different filter operations (**Figure 2A**). Next, the motion map indicated the temporal depth change around the current frame for each pixel (**Figure 2B**) by approximating the first derivative relative to the noise.

From the motion map, the 3D signal was derived as a 1D value representing the movement activity for each frame (**Figure 2C**). The higher the values of the pixels indicating movement, the higher the resulting 3D signal. Thus, the 3D signal depended on the number of included pixels and their motion response. Next, peaks of the 3D signal were categorized in actual movements or peaks induced by noise. We used two thresholds to identify real movements: one to define start and stop of a potential movement separating floor noise from peaks, and the other identified real movements and ignored noise induced peaks. Threshold calibration was performed for each room individually.

In a next step, the annotated movements were classified into RMs and non-RMs. As RMs occur rhythmically, 3D signals of annotated movements was analyzed using fast Fourier transform (FFT) with the goal of finding characteristics separating the classes in the frequency domain. FFT used a window of 3 s to achieve a resolution of 0.33 Hz according to the time–frequency uncertainty. We used an overlap of 50% and zero padding, resulting in segments of 1.5 s. Due to padding, possible inaccuracies may be induced at the first and the last segment of a movement, as detected by our algorithm; therefore, we excluded these segments from the analyses. RM segments showed a narrow (∼2 Hz) frequency band, with the mean frequency representing the participant’s movement frequency (**Figure 2D**). We also detected an absence of frequency components in the range below this peak. In contrast, general movements presented a blurred spectrum suggesting an overlay of many frequencies. From this information, features from the frequency domain were engineered with the aim of identifying spectral characteristics of RMs distinct from those of non-RMs. The best features for separation and their thresholds were identified using Cross-Validation and Random Decision Forests (23). The final implementation used the three best features for classification: the power of the lower frequencies (0–0.67 Hz) best separated the two movement types followed by the frequency with the highest power and the frequency with the second highest power.

**Figure 3:**
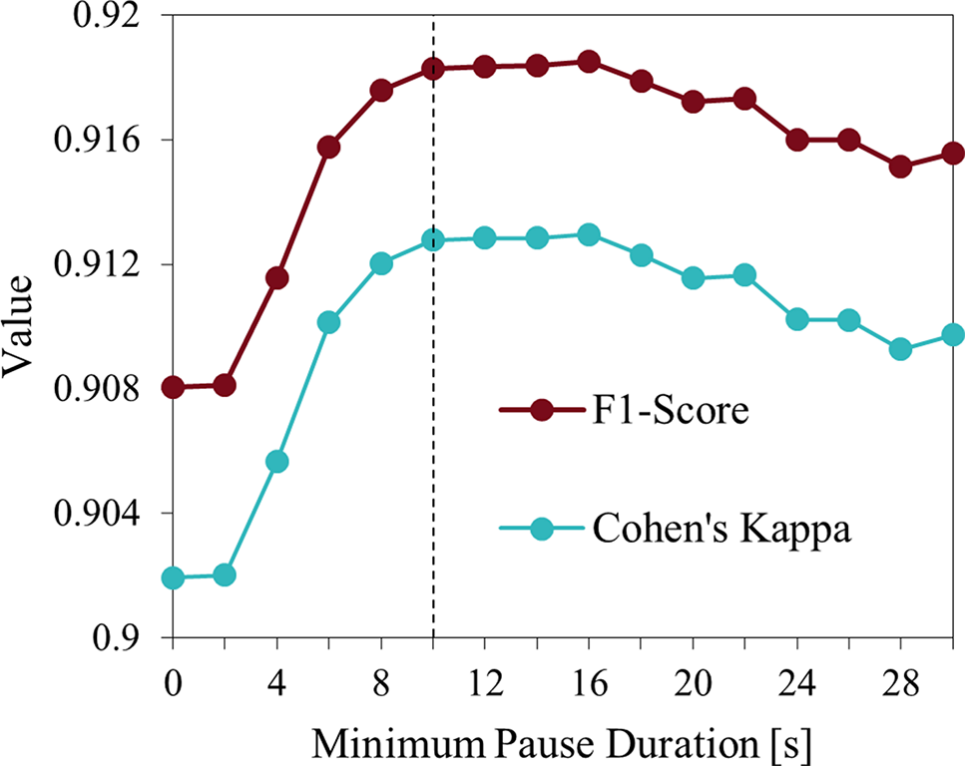
Minimum pause duration influence on evaluation measures. Indicated are the F1-score and Cohen’s kappa for different values of the minimum pause duration applied to 3D video and visual-manual method. Durations >10 s do not result in further improvements.

As noted in 2.2, when annotating the ground truth, termination of a RM episode was originally defined as a cessation of movement lasting for at least the duration of two movement cycles. In practice, for a 2 Hz movement frequency, this is equivalent to only 1 s. As it was not possible to count movements with sufficient precision to apply the same rule for 3D annotations, we introduced a revised threshold minimum pause duration that was independent of movement frequency. Specifically, a minimum pause duration of 10 s was based on both pragmatic and clinical considerations. Evaluation of this 10 s rule resulted in F1-score and Cohen’s kappa with highest agreement when applied to the 3D and the manual method (**Figure 3**). This finding suggested that the previously defined rule was too sensitive to apply when annotating manually. The new 10 s rule was also applied to manual annotations.

### Algorithm Evaluation

Comparison of automatic 3D and manual 2D approaches used the following standard classification features: false positive rate, true positive rate, Cohen’s kappa or F1-score, and comparison of indices such as number of RM episodes or episode duration. Even though many single events were recorded, due to the low number of recorded nights and participants, we chose not to include measures of statistical significance in our results.

### Severity Measures for RMD

Clinically relevant indexes of movement severity were defined as follows:

1. Total duration of rhythmic episodes based on time in bed (s)
2. Total duration of non-rhythmic episodes based on time in bed (s)
3. Number of RM episodes based on time in bed (count)
4. Mean duration of RM episodes (s)
5. Rhythmic movement index (RM index)* (episodes/h of time in bed)
6. Duration index* (% of time in bed occupied by RMs)
7. Frequency index*(Hz)

Furthermore, we developed graphical plots to map these indices to time of night:

1. RM time of night distribution plot
2. Fragmentation plot*
3. Duration distribution plot*
4. Frequency distribution plot*

Measures/plots marked with an asterisk (*) are novel and aim to improve objective RMD assessment. They are based on indices used to characterize other sleep disorders like the PLM index or AHI (22, 24) both calculated as numbers of events *per* hour of sleep.

To maximize detection of RMs, we purposively excluded neurophysiological measures of the sleep-wake state, so our calculations such as the RM index were based on time in bed. The RM index (Equation 1) therefore reflects how often the night is disrupted by RM episodes and includes RMs after lights out before sleep onset.

(1) $$RM\;index\;=\;\frac{number\;of\;RMs\;(episodes)}{time\;in\;bed\;(h)}$$

The fragmentation plot illustrates the distribution of time between consecutive RM episodes. It is based on the concept of the inter-movement-interval (imi) histogram as used in PLM research (25).

The RM index and plot alone may not be sufficient to describe RMD severity. In RMs, the effort and amplitude of movements is independent of the number of episodes. However, the duration of the episodes provides an indication of severity. For example, 40 × 2 s duration RM episodes would generate the same RM index as 40 × 3-minute episodes if the episodes start at the same times. Clearly, the second example expends more movement effort due to longer episode duration. Therefore measurements that reflect this difference in severity are essential for RMD diagnosis. To address this dimension, we introduced the duration index (Equation 2) calculated as the ratio of RM time to total time in bed. 

(2) $$duration\;index=\frac{RM\;time\;(h)}{time\;in\;bed\;(h)}$$

The corresponding duration distribution plot provides additional information. With these novel indices, RM severity was characterized through combining variables of sleep fragmentation and duration.

To provide further characterization of RM episodes, we propose the frequency index. This index provides more detailed insight on effort, thereby being a valuable extension of the duration index. The duration index already indicates how much time is spent in RMs but does not capture the mechanical effort in terms of how often the individual actually moved during this time. Slow RMs require less physical effort than faster ones since a change of movement direction occurs much less often. The frequency index reflects this movement effort by calculating the mean RM frequency (Equation 3 with k, the total number of episodes, and , the mean frequency of the recording’s n-th RM episode).

(3) $$frequency\;index\;=\;\frac{\sum_{n=1}^{k}{\dot{f}}_{n}}{k}$$

Furthermore, the frequency distribution plot indicates time of the night when specific frequencies occurred. Thus, the somnologist can observe how RM frequency, and thus effort, changes over the night. Automatic 3D with its ability to rapidly process complex data offers significant advantages over cumbersome manual 2D video scoring to generate such frequency-based data.

In summary, we propose three new indices for harmonized RMD assessment. The RM index reports how often a night is disturbed by RM episodes; the duration index gives an indication on how much effort is involved in RMD episodes, and finally, the frequency index provides data on the mean frequency of RMs.

## Results

Six children with sleep-related RMD between age 5 and 14 years (M_age_: 9.0 years, SD: 4.2 years) were recruited in the UK, of whom two were female and four male. Recorded movement semiologies included head banging, head rolling, body rocking, and body rolling. One participant did not exhibit RMs during the study despite video documentation of movements in the home environment.

**Table 1** compares scoring results for automatic 3D video versus visual-manual 2D analysis. Records of individual results are shown in **Table S1** of the Appendix section. As expected, most of the segments in each record were classified as negative. Therefore, the resulting true negative rate and accuracy were higher or equal to 99%. Segments of incorrect episode classification comprise a small proportion compared to the number of true episode classifications. A good performance is indicated by an F1-Score of 0.918 calculated from the Positive Predictive Value of 0.912 and the True Positive Rate of 0.924. Some records show even higher values. What also stands out in the table is the value of Cohen’s kappa of ∼0.9 stating almost perfect agreement of the two methods, according to Landis and Koch (26).

**Table 1:** Classification results of the automatic 3D video method versus manual approach across 12 nights of data.

Performance metric	Value
Number of true negative segments*	148,669
Number of false positive segments*	894
Number of false negative segments*	762
Number of true positive segments*	9,281
Number of segments classified as RM by ground truth*	10,043
Number of segments classified as non-RM by ground truth*	149,563
True positive rate**	0.924
True negative rate**	0.994
False negative rate**	0.076
False positive rate**	0.006
Positive predictive value**	0.912
Accuracy**	0.990
F1-score**	0.918
Cohen’s kappa**	0.913

(*) Values were derived as sum of all individual records. (**) Values were calculated with classification scores marked with (*).

**Table 2** shows the evaluation results on RMD measures. Presented are the mean values over all recordings. Results for individual participants are given in **Table S1** of the appendix section. The most interesting aspect of **Table 2** is the RM duration. While automatic 3D video scoring finds a mean duration of 0.71 h *per* participant, the manual 2D approach shows a mean duration of 0.70 h *per* patient, a difference of 36 s. The average number of episodes detected by 3D is 28.92 *per* time in bed, while the manual approach detects only 9.17 on average. Since the mean duration is nearly the same but number of detected episodes differs, the results show that 3D tends to discriminate discrete episodes more sensitive. Episodes detected with the manual approach have a mean duration of 205.70 s, which is more than double of that detected by 3D analysis. Our newly introduced indices reflect these findings well: The average duration index is equal for both methods with a value of 0.06. The RM index is augmented for 3D with a value of 2.60 compared to 0.77 for the manual approach.

**Table 2:** Evaluation results for automatic 3D video analysis and visual-manual 2D annotations separately.

	3D	Manual
RM duration (h)	0.71	0.70
Non-RM duration (h)	10.37	10.38
Number of episodes	28.92	9.17
Mean episode duration (s)	73.85	205.79
RM index (episodes/h)	2.60	0.77
Duration index (%)	6.04	5.93
Frequency index (Hz)	1.08	
Bed time (h)	11.08	11.08
Total time (h)	132.99	132.99

Presented are the mean RMD measures over all recordings.

In addition to the numeric scores, RM episodes can be plotted graphically using our novel indices.

**Figure 4:**
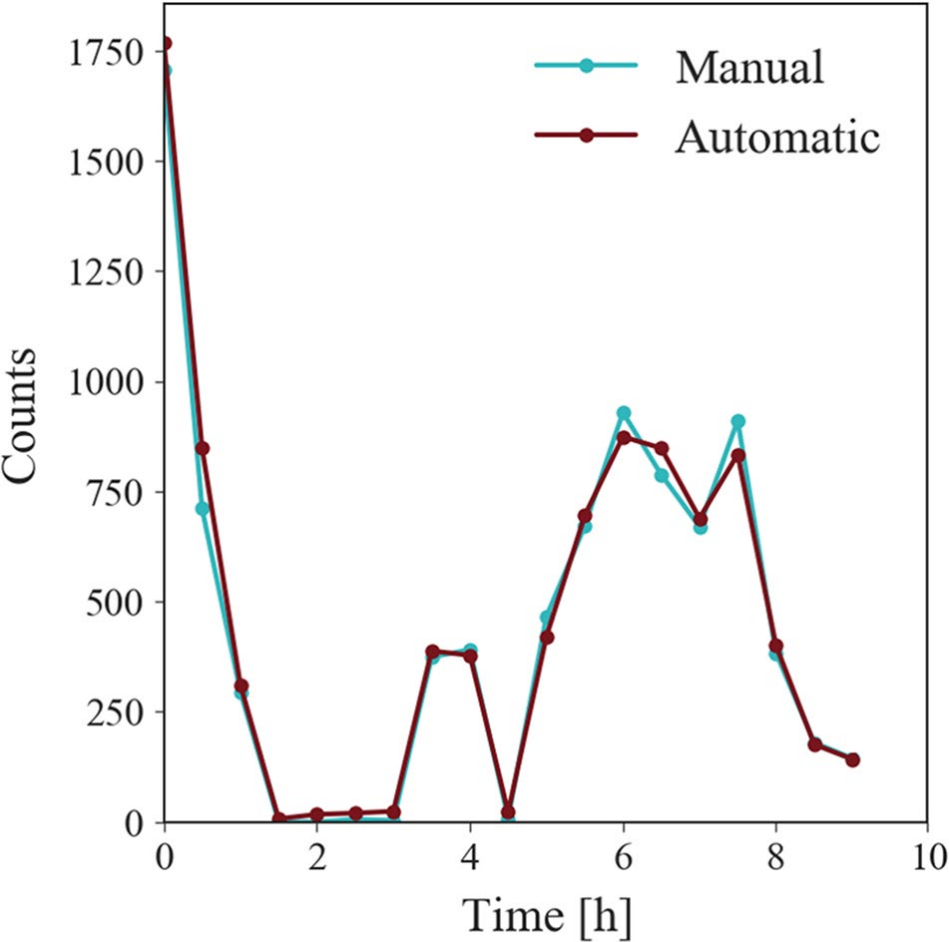
RM time of night distribution plot for all recordings. Each data point shows how many RM episodes occur over all recordings in time intervals of 30 min. RMs occur predominantly in the beginning of the night and the start of the second half of the night. Automatic 3D finds high agreement with data obtained by manual annotations.

First, **Figure 4** shows the distribution of RMs over the night as the accumulated sum of all recordings. The y-axis shows the counts of RMs occurring in time intervals of 30 min. The figure shows that RMs are more likely to occur at the beginning of the night and then augment at the beginning of the second half of the night. From this data, we can also see that automatic 3D and manual 2D annotations highly agree.

**Figure 5:**
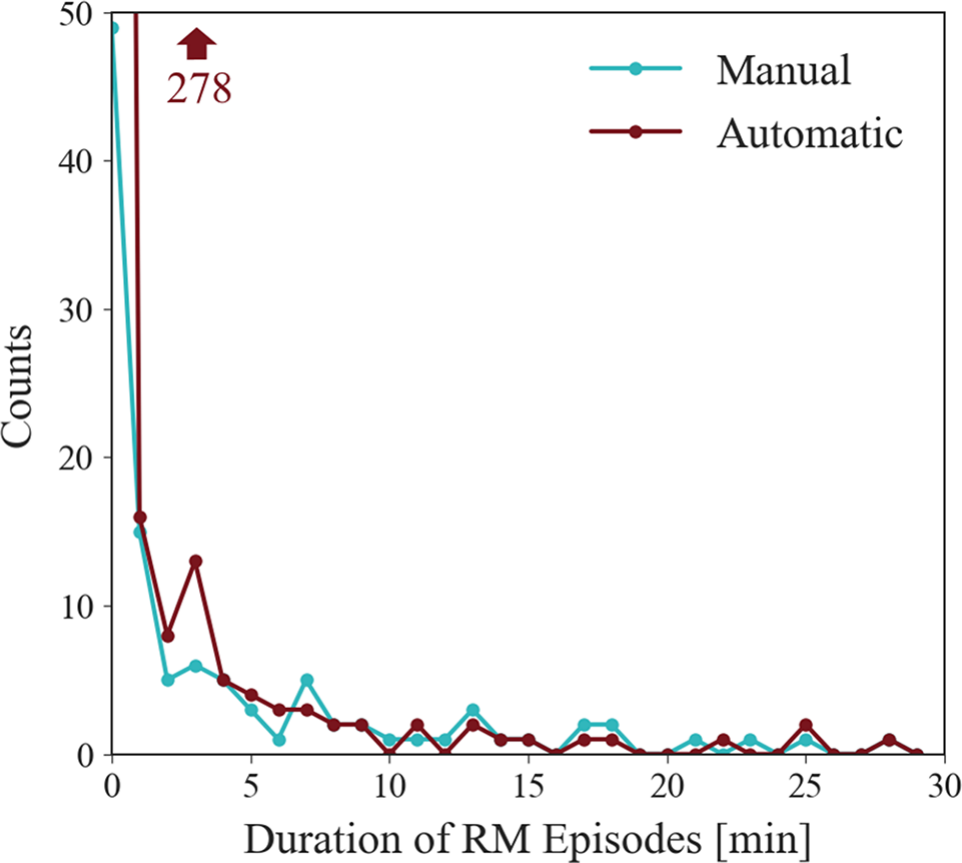
Duration distribution plot for all recordings. Represented is the count of detected RMs with a duration between intervals of 1 min. Noticeable is the high count of durations between 0 and 1 min. The data point for automatic 3D in this first interval exceeds the plot window, but its value is indicated by the arrow.

**Figure 5** shows the RM episode durations for automatic 3D and manual 2D approaches. Interestingly we observed that the majority of events had a duration of less than 1 min. Importantly, 3D detected more episodes of shorter duration.

**Figure 6:**
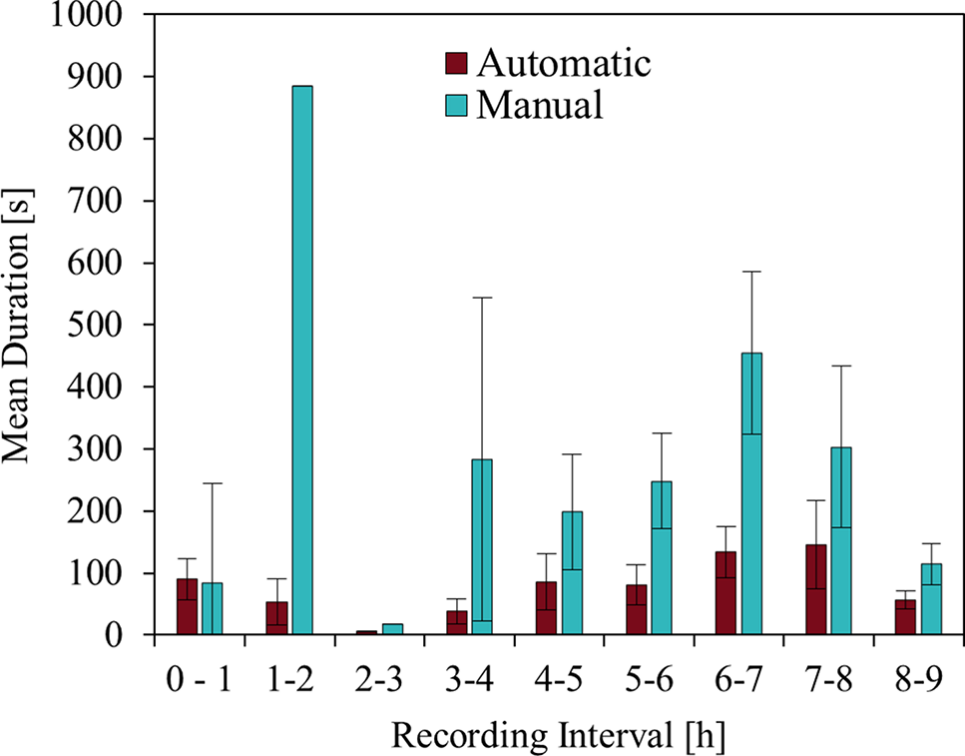
RM duration distribution overnight. The plot shows the mean duration of RMs per hour of the recorded nights for the automatic 3D and manual 2D method. Error bars indicate the standard error of the mean. RM durations indicate high variety for each of the periods. Periods 1-2 and 2-3 include only a single RM each for the manual data, where the standard deviations equal zero.

Analysis on duration distribution over the night shows that durations largely vary over most periods of the night (**Figure 6**). During sleep onset, RMs show longer durations, while results show that following periods are of shorter duration. Periods 1-2 and 2-3 include only one movement each for the manual data. With the start of the second half of the night, movement durations augment whereby movements in the last period again diminish to medium durations.

**Figure 7:**
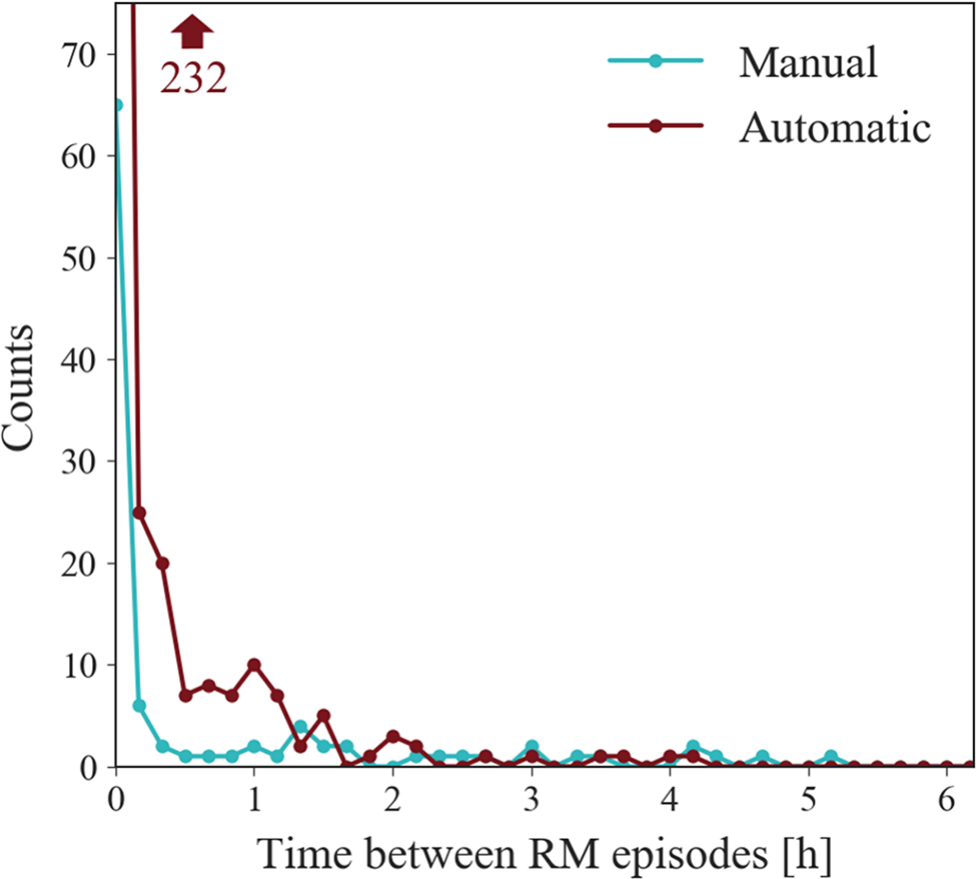
Fragmentation plot of all records. Illustrates the distribution of time elapsed between onset of consecutive RMs presented with a convenience interval width of 10 min. 3D reports higher fragmentation than the manual approach as the majority of time periods are less than 10 min. This data point lies outside of the plot window, but its value is indicated by the arrow.

The fragmentation plot, **Figure 7**, reflects the time interval between consecutive RM episodes from the start of the movement to the start of the subsequent one and indicates sleep quality. The episode duration plot and the higher number of 3D detected episodes predict the findings of the fragmentation plot, namely, that 3D reports a higher fragmentation than manual annotation, also supported by the higher RM index.

**Table 3** compares three individual participants’ records using the proposed RMD indices, illustrating how this allows rapid comparison between cases and illustrates the spectrum of severity of RMD. We chose these three examples since they best represent the spectrum of severity.

**Table 3:** Proposed indices on recordings showing different manifestations of RM symptoms.

Case	Duration index (%)	Rhythmic movement index (episodes/h)	Frequency index (Hz)
A	0.08	0.59	0.87
B	15.88	2.41	1.05
C	5.88	2.16	0.97

Chosen recordings were obtained during adoption nights.

**Figure 8:**
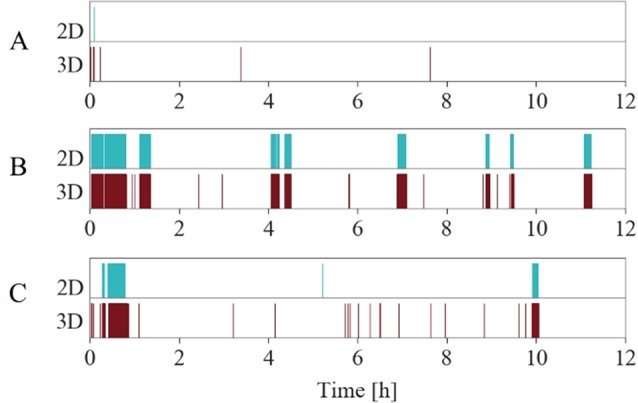
RM time of night distribution plots according to **Table 3**. The three examples show automatic 3D annotations (lower, red) and manual 2D annotations (upper, cyan) over time. RM annotations are indicated with a colored rectangle. Periods without annotations stay blank. Case A only shows few RMs in the beginning of the night, hardly recognizable on the full night scaled plot. Case B exhibits the most severe form of RMD, while case C exhibits mild symptoms compared to B.

RM distribution across the night (**Figure 8**), generated by both automatic 3D and manual 2D annotations for the cases, graphically represents both the duration index and RM index reported in **Table 3**. Case A has a duration index close to zero, suggesting relatively mild RMD as shown in the plot. Additionally, the RM index is very low. The frequency index does not add any value in this case since the duration and number of RM episodes are low. Case B illustrates greater severity. This is again reflected in the indices in **Table 3**, where the duration index indicates that 15.88% of the total night is spent in RMs. The RM index of 2.41 indicates that each hour of the night is disturbed more than twice by an RM episode. Finally, case C shows an intermediate severity. Again, the proposed indices reflect this estimation perfectly. Note how the frequency index yields additional useful information: Not only does participant B have RMs for a longer period of the night and shows more sleep fragmentation but also the higher frequency of movements denotes greater effort engaged in RMs across the night.

**Figure 9:**
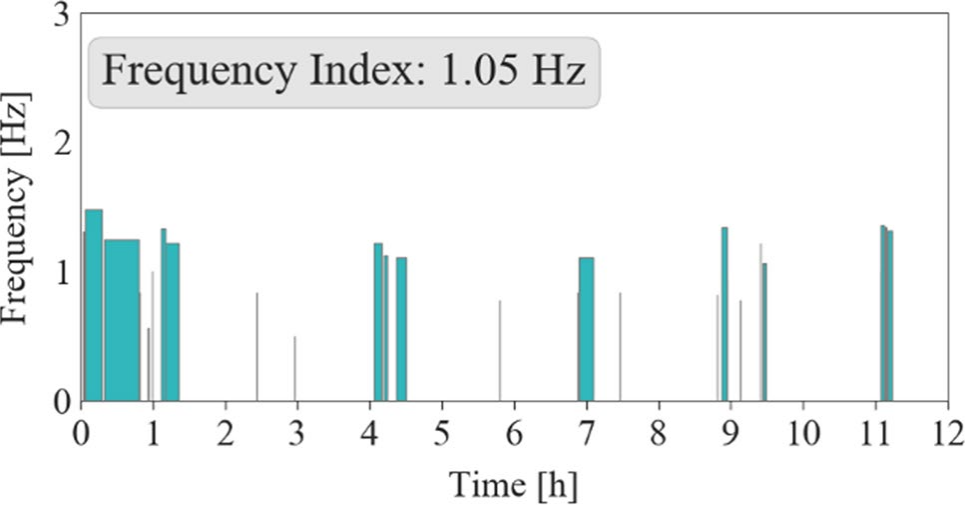
Frequency distribution plot of an individual record (Case B). The mean frequency of an episode is plotted over the time in hours. For this case, frequency remains relatively stable over the night.

In addition to the measures already described, **Figure 9** shows an example of frequency analyses from the 3D data of one individual. The figure is similar to the RM episode distribution plot where episodes are plotted over time. In contrast, the y-axis represents the mean frequency for each episode, illustrating relative stability of movement frequency across the night.

## Discussion

### Benefits of Automatic 3D Analysis

The objective of this work was to develop and evaluate an automated method to quantify RMs using a contactless 3D video approach. Furthermore, we aimed to define useful indices to quantify RMD severity to provide information for the development of future international scoring standards.

Obvious improvements were introduced by replacing manual analysis of 2D videos by automatic and marker free 3D video analysis. Annotating 2D videos manually is time-consuming and monotonous to perform, and measures such as movement frequency are difficult to determine. In practice, annotating several hours of data cannot be performed with the same reliability and speed as computerized approaches.

This work also set out to propose new measures to better characterize severity of RMD in the same way that the apnea-hypopnea-index or the periodic limb movement index is used to describe severity of sleep apnea and PLM disorder, respectively. Based on this concept, we introduced the RM index, reflecting how often the time in bed is disrupted by RM episodes. This index is in analogous to the periodic leg movement index and makes use of the same formula. However, established frequency count indices fail to capture the proportion of the time occupied by RMs, so a measure of episode duration was also developed: Longer periods of movement expend greater effort and result in more sleep disruption. Graphical plots of RM distribution through the night, frequency changes, duration, and fragmentation add value by helping to objectively quantify RMD through the sleep period. Better understanding of RMD severity using objective and reproducible quantitative parameters has the potential to support clinical diagnosis and determine at what threshold RMs become a disorder. The ICSD III criterion D refers to disturbance of sleep, but precisely what threshold of sleep fragmentation or duration of RM is important has yet to be determined. Such quantitative measures would facilitate the study of the relationship between RMD severity and impact on health, cognitive, behavioral, and quality of life measures. They also provide a means of measuring treatment outcomes in both clinical practice and treatment trials.

Automatic detection of RMs also creates the possibility for real time intervention treatment, whereby the automated 3D system triggers the onset/offset of connected devices. A number of authors have suggested treatment approaches that include, for example, aversion or stimulus substitution approaches (5, 27, 28), which rely on a response to initiation of movement. For example, the Somnomat rocking bed, which formed part of this feasibility study (19), could be activated in response to the patient’s RMs.

### Differences Between Automatic 3D and Manual 2D Analysis Methods

The most notable difference between automatic 3D and visual-manual 2D analysis was in RM episode counts. 3D was more sensitive in discriminating episodes than 2D, as short movement pauses may be overlooked by manual scoring. Our experience suggested merit in defining discrete episodes of RMs with a minimum inter-movement duration of 10 s. This criterion was simpler to operationalize rather than a movement frequency-based criterion (defined for manual annotations as equal to or greater than the duration of two RMs), which will differ between individuals. This definition might even improve interrater variability in manual scoring. However, it should be recognized that this 10 second criterion was generated pragmatically for the purposes of this study and should be tested in future studies.

The automatic approach classified some movements as RMs that were not identified by manual scorers. False positive detections occurred when 3D detected a general movement having rhythmic elements. Similarly, RMs with a slow general movement in parallel resulted in some false negative scoring. In these cases, Fourier analysis yielded a frequency spectrum that appeared similar to that of a general non-RM. However, falsely classified movements were rare compared to the number of correctly classified ones. From a practical clinical and research stand point, automated analysis with the level of accuracy generated by our data offers significant advantages over manual scoring and, importantly, the contactless nature of the measurement device allows also RMs to be assessed without inhibiting movements.

### Limitations of This Study

The study included only six participants analyzed over two nights. One participant did not show RMs during any of the two nights. The methodologies should be replicated in future datasets. Intra- and inter-scorer variability is not available because annotations were revised by the same scorer.

## Conclusion

We present a novel approach to characterize RMD in children using contactless automatic 3D video assessment. This method not only turned out to be fast and highly reliable but also matched the results of manual scoring to a high degree. Three novel indices are proposed: the duration index, the RM index, and the frequency index, that offer a clinically relevant multi-dimensional quantification of RM severity and lay the foundation for a systematic approach to assessment of this poorly understood sleep disorder. This novel use of readily available 3D technology offers a practical measurement approach that could be used in the laboratory or home environment. In conclusion, automatic 3D has the potential to become a valid method to assess RMD severity in children and to enable comparable and reproducible quantification of RM episodes.

## Data Availability Statement

The datasets for this study will not be made publicly available because 3D and 2D video data shows participant identifying information.

## Ethics Statement

This study was carried out in accordance with the Declaration of Helsinki. The UK National Research Ethics Committee and Health Research Authority (IRAS 234505), the Cantonal Ethics Committee of Zurich (KEK 2017-01880) and Swissmedic (2017-MD-0035) approved the study. Informed consent was obtained from the responsible adults and subjects gave informed assent.

## Author Contributions

MG, RS, EW, QR, LJ, PA, RR, and CH conceived, planned, and carried out the study and measurements. MG, RS, H-PL, OJ, HG, and CH supervised the project. MG, BK, and CW designed the computational framework and algorithms. MG and HG analyzed the data. MG wrote the manuscript with support from BK, CW, RS, HG, and CH. All authors provided feedback and helped shape the research and critically reviewed the final manuscript.

## Funding

The work of MG is supported by the Österreichische Forschungsförderungsgesellschaft (8060159) as part of a dissertation funding. A Global Partnership Award from the Univesity of Southampton, UK provided funding to enable planning meetings between collaborators, analysis of 3D video data and travel of UK participants to Zurich. The National Science Foundation (Grant CR3213-162809/1) and the Commission for Technology and Innovation (Grant 17988) supported infrastructure and salaries.

## Conflict of Interest

The authors declare that the research was conducted in the absence of any commercial or financial relationships that could be construed as a potential conflict of interest.
