# Supplementary material for: A Novel Approach to Assess Sleep-Related Rhythmic Movement Disorder in Children Using Automatic 3D Analysis
Source: Front Psychiatry. 2019 Oct 16;10:709. doi: 10.3389/fpsyt.2019.00709 (PMC6806394; doi:10.3389/fpsyt.2019.00709)
Supplement: Supplementary file 1 [file DataSheet_1.docx]

# Appendix

## Kinect Sensor

Each image has an initial resolution of 512x424 pixels. The sensor emits weak, amplitude-modulated incoherent near-infrared light pulses at a wave-length of 860 nanometers. Surfaces in the scene reflect the light, further measured by a matrix of detector diodes inside the camera. The electronic circuits behind each pixel provide both grey scale values and the time-of-flight of the near-infrared light resulting in a 2D infrared image and a 3D depth image.

## Record individual results

Table S1 shows record individual results for RMD measures and classification scores.

Table S1: Record individual results for RMD measures and classification scores.

| **Rec_id** | **3D_RM_duration [sec]** | **Anno_RM_duration [sec]** | **3D_nonRM _duration [sec]** | **Anno_nonRM _duration [sec]** | **3D_EPS [counts]** | **Anno_EPS [counts]** |
| --- | --- | --- | --- | --- | --- | --- |
| RM133001 | 182.36 | 53.00 | 50089.64 | 50219.00 | 26 | 6 |
| RM133003 | 1155.07 | 914.02 | 41324.93 | 41565.98 | 40 | 20 |
| RM153001 | 29.67 | 0.00 | 36346.33 | 36376.00 | 6 | 0 |
| RM153002 | 67.40 | 0.00 | 34972.60 | 35040.00 | 9 | 0 |
| RM164001 | 6399.14 | 6389.22 | 33925.86 | 33935.78 | 27 | 12 |
| RM164002 | 6724.20 | 6767.77 | 35575.80 | 35532.23 | 31 | 12 |
| RM179001 | 1021.27 | 838.34 | 31337.73 | 31520.66 | 42 | 3 |
| RM179003 | 168.93 | 9.00 | 36093.07 | 36253.00 | 26 | 1 |
| RM185001 | 1065.16 | 1057.51 | 38277.84 | 38285.49 | 23 | 9 |
| RM185002 | 11037.23 | 11741.00 | 34841.77 | 34138.00 | 56 | 39 |
| RM197001 | 2351.06 | 2043.89 | 37640.94 | 37948.11 | 24 | 4 |
| RM197003 | 436.13 | 402.00 | 37705.87 | 37740.00 | 37 | 4 |
|  |  |  |  |  |  |  |
| **Rec_id** | **3D_mean_EPS _duration [sec]** | **Anno_mean_EPS _duration [sec]** | **3D_rhyhtmic_ movement_idx** | **Anno_rhythmic_ movment_idx** | **3D_Duration _idx** | **Anno_Duration _idx** |
| RM133001 | 7.01 | 8.83 | 1.86 | 0.43 | 0.00 | 0.00 |
| RM133003 | 28.88 | 45.70 | 3.39 | 1.69 | 0.03 | 0.02 |
| RM153001 | 4.94 |  | 0.59 | 0.00 | 0.00 | 0.00 |
| RM153002 | 7.49 |  | 0.92 | 0.00 | 0.00 | 0.00 |
| RM164001 | 237.01 | 532.44 | 2.41 | 1.07 | 0.16 | 0.16 |
| RM164002 | 216.91 | 563.98 | 2.64 | 1.02 | 0.16 | 0.16 |
| RM179001 | 24.32 | 279.45 | 4.67 | 0.33 | 0.03 | 0.03 |
| RM179003 | 6.50 | 9.00 | 2.58 | 0.10 | 0.00 | 0.00 |
| RM185001 | 46.31 | 117.50 | 2.10 | 0.82 | 0.03 | 0.03 |
| RM185002 | 197.09 | 301.05 | 4.39 | 3.06 | 0.24 | 0.26 |
| RM197001 | 97.96 | 510.97 | 2.16 | 0.36 | 0.06 | 0.05 |
| RM197003 | 11.79 | 100.50 | 3.49 | 0.38 | 0.01 | 0.01 |
|  |  |  |  |  |  |  |
| **Rec_id** | **3D_Frequency _idx** | **bed_time [h]** |  |  | **positive** | **negative** |
| RM133001 | 0.93 | 13.96 |  |  | 19 | 16740 |
| RM133003 | 1.39 | 11.80 |  |  | 307 | 13854 |
| RM153001 | 0.87 | 10.10 |  |  | 0 | 12127 |
| RM153002 | 0.79 | 9.73 |  |  | 0 | 11681 |
| RM164001 | 1.05 | 11.20 |  |  | 2097 | 11346 |
| RM164002 | 0.99 | 11.75 |  |  | 2257 | 11844 |
| RM179001 | 0.99 | 8.99 |  |  | 279 | 10509 |
| RM179003 | 0.89 | 10.07 |  |  | 3 | 12086 |
| RM185001 | 1.48 | 10.93 |  |  | 351 | 12765 |
| RM185002 | 1.72 | 12.74 |  |  | 3916 | 11378 |
| RM197001 | 0.97 | 11.11 |  |  | 681 | 12651 |
| RM197003 | 0.94 | 10.60 |  |  | 133 | 12582 |
|  |  |  |  |  |  |  |
| **Rec_id** | **tn** | **fp** | **fn** | **tp** | **tpr** | **tnr** |
| RM133001 | 16680 | 60 | 19 | 0 | 0.00 | 1.00 |
| RM133003 | 13744 | 110 | 38 | 269 | 0.88 | 0.99 |
| RM153001 | 12117 | 10 | 0 | 0 |  | 1.00 |
| RM153002 | 11658 | 23 | 0 | 0 |  | 1.00 |
| RM164001 | 11279 | 67 | 69 | 2028 | 0.97 | 0.99 |
| RM164002 | 11793 | 51 | 67 | 2190 | 0.97 | 1.00 |
| RM179001 | 10364 | 145 | 86 | 193 | 0.69 | 0.99 |
| RM179003 | 12026 | 60 | 3 | 0 | 0.00 | 1.00 |
| RM185001 | 12727 | 38 | 30 | 321 | 0.91 | 1.00 |
| RM185002 | 11266 | 112 | 348 | 3568 | 0.91 | 0.99 |
| RM197001 | 12528 | 123 | 22 | 659 | 0.97 | 0.99 |
| RM197003 | 12487 | 95 | 80 | 53 | 0.40 | 0.99 |
|  |  |  |  |  |  |  |
| **Rec_id** | **fnr** | **fpr** | **ppv** | **acc** | **f1** | **ckappa** |
| RM133001 | 1.00 | 0.00 | 0.00 | 1.00 | 0.00 | 0.00 |
| RM133003 | 0.12 | 0.01 | 0.71 | 0.99 | 0.78 | 0.78 |
| RM153001 |  | 0.00 | 0.00 | 1.00 |  | 0.00 |
| RM153002 |  | 0.00 | 0.00 | 1.00 |  | 0.00 |
| RM164001 | 0.03 | 0.01 | 0.97 | 0.99 | 0.97 | 0.96 |
| RM164002 | 0.03 | 0.00 | 0.98 | 0.99 | 0.97 | 0.97 |
| RM179001 | 0.31 | 0.01 | 0.57 | 0.98 | 0.63 | 0.61 |
| RM179003 | 1.00 | 0.00 | 0.00 | 0.99 | 0.00 | 0.00 |
| RM185001 | 0.09 | 0.00 | 0.89 | 0.99 | 0.90 | 0.90 |
| RM185002 | 0.09 | 0.01 | 0.97 | 0.97 | 0.94 | 0.92 |
| RM197001 | 0.03 | 0.01 | 0.84 | 0.99 | 0.90 | 0.90 |
| RM197003 | 0.60 | 0.01 | 0.36 | 0.99 | 0.38 | 0.37 |
